# Zinc Deficiency Presenting With Diverse Symptoms in a Young Patient: A Case Report

**DOI:** 10.7759/cureus.66034

**Published:** 2024-08-02

**Authors:** Masatoshi Inoue

**Affiliations:** 1 Nephrology, Tsushima City Hospital, Aichi, JPN

**Keywords:** zinc deficiency, iron-deficiency, immunity impairment, glucose intolerance, anemia

## Abstract

Despite its prevalence, zinc deficiency often goes undiagnosed due to nonspecific symptoms. This study examined the case of an 18-year-old woman who presented with urinary tract infection, anemia, and insulin dysfunction and was ultimately diagnosed with zinc deficiency. Oral zinc supplementation significantly improved the patient's condition. Zinc is essential for the activity of numerous enzymes and affects immune function, protein structure, and endocrine regulation, but the cause is often unknown because symptoms and data abnormalities are nonspecific. The patient's diet was high in foods that inhibited zinc absorption, likely exacerbating the deficiency. This case illustrates the importance of considering zinc deficiency in patients with diverse and unexplained symptoms. Prompt recognition and treatment with zinc supplementation can lead to rapid and complete recovery. We hope that this case will contribute to the future diagnosis of zinc deficiency for clinicians.

## Introduction

Zinc is required for the activity of >300 enzymes, and its presence in proteins may be directly involved in chemical catalysis or may be important for maintaining protein structure and stability [1]. Zinc deficiency is widespread and affects systemic growth, metabolism, immune response, endocrine regulation, hematopoiesis, and the development of connective tissue, bone, and teeth [2-3]. Zinc deficiency is a prevalent condition among the elderly and children, but it is relatively uncommon among young adults. The resulting symptoms and data abnormalities are nonspecific, making a zinc deficiency challenging to diagnose. Currently, there are no established diagnostic criteria for this deficiency commonly used worldwide. This study reports the case of a young patient who presented to the hospital with a urinary tract infection, impaired insulin function, and anemia. She was diagnosed with zinc deficiency and successfully treated with oral zinc supplementation. We hope that this report will assist in the accurate diagnosis of a previously overlooked zinc deficiency.

## Case presentation

An 18-year-old woman presented to the emergency department with a fever and general malaise. She had a history of anemia, which was treated with sodium ferrous citrate at a gynecologist’s office for about one year. Her consciousness was lucid, body temperature was 41.1℃, blood pressure was 84/37 mmHg, respiratory rate was 18 breaths per minute, heart rate was 133 beats per minute, and oxygen saturation was 98% (room air). In the emergency department, the patient appeared to be in no acute distress with a normal physical examination. Chest X-ray and whole-body computed tomography were unremarkable. Her serum hemoglobin level was decreased (88 g/L; normal 137-168), and her C-reactive protein level was elevated (4.4 mg/dL; normal 0-0.3) (Table 1).

**Table 1:** Laboratory investigations for this patient Note: ND=not done

	Reference range	Admission	Day 1	Day 2	Day 3	Day 6	Day 26	Day 47	Day 96	Day 145
Hemoglobin, g/L	116-148 g/L	88	91	88	81	81	108	116	117	127
Hematocrit, %	35.1-44.4 %	27.5	29.2	29.5	27.9	27	33.6	35.9	34.7	36.6
White blood cell count, ×10^9^/L	3.0-8.6×10^9^/L	5.3	4.5	5.8	4.1	3.2	4	5.7	5.2	6.5
Neutrophil count, ×10^9^/L	1.8-7.5×10^9^/L	4.1	3.2	3.3	1.6	1	2.1	3.4	2.7	4.5
Lymphocyte count, ×10^9^/L	1.0-4.8×10^9^/L	0.86	0.99	1.8	1.8	1.4	1.4	1.7	1.9	1.5
Platelets, ×10^9^/L	158-348×10^9^/μL	232	237	202	219	215	204	257	209	201
Creatinine, μmol/L	46-79 μmol/L	80	82	64	60	51	57	64	64	65
Zinc, μg/dL	65-110 μg/dL	ND	30	ND	ND	ND	175	203	62	62
Copper, μg/dL	66-130 μg/dL	ND	187	ND	ND	ND	93	106	171	193
Ferritin, ng/mL	3.4-89 ng/mL	ND	575	ND	ND	ND	92.5	ND	4.7	56.3
Alkaline phosphatase, U/L	38-113 U/L	ND	ND	ND	ND	40	64	61	46	52
Glucose, mg/dL	70-109 mg/dL	110	95	74	162	100	145	89	106	97
IgG, g/L	8.6-17.4 g/L	ND	ND	7.07	ND	ND	6.89	7.43	7.24	7.97
IgA, g/L	0.93-3.93 g/L	ND	ND	0.82	ND	ND	0.8	0.79	0.82	0.83
IgM, g/L	0.5-2.7 g/L	ND	ND	1.36	ND	ND	1.25	1.29	1.27	1.2
Urine ketones	-	1+	4+	4+	4+	-	-	-	-	-
Note: ND=not done										

**Figure 1:**
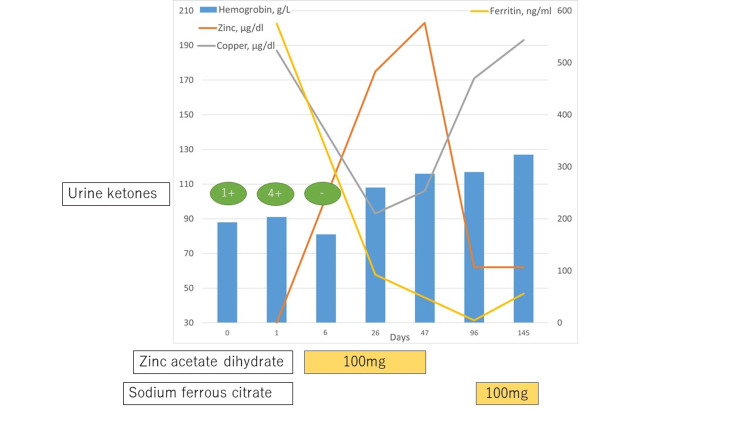
Time course of this patient

Her urine leukocytes were 3+ (urine sediment was not observed due to oliguria) and urine ketones were 1+. Blood cultures and multiple nasal swab PCR tests for COVID-19 were all negative. We admitted her and started treatment with 3 liters of intravenous fluids over the next 24 hours and ceftriaxone 2 g/day, but her urine ketones increased to 4+ on day 1. The results of the laboratory tests taken on day 1 were known on day 5. Because her serum zinc level was significantly decreased (30 μg/dL; normal 65-110), we started her on an oral dose of 100 mg/day of zinc acetate dihydrate. Urine ketones became negative and anorexia also improved on day 6, so antimicrobial therapy was stopped. Her bone mineral density and physical growth were normal. All symptoms resolved and she was discharged seven days after admission. She ate only pantries and milk for lunch and dinner to save time as she was a student taking entrance exams. We instructed her to eat a balanced diet, with an emphasis on seafood and meat. Her serum hemoglobin was elevated to 103 g/L and her zinc level to 175 μg/dL on day 23. We discontinued zinc acetate dihydrate on day 47. Her serum ferritin level was decreased to 4.7 ng/mL on day 96, so we administered sodium ferrous citrate 100 mg/day to her. All laboratory abnormalities improved, and she felt lighter and could concentrate better than before. She is under regular follow-up without recurrence (Figure 1).

## Discussion

In this article, we describe a female patient diagnosed with severe zinc deficiency who had a urinary tract infection, anemia, and insulin dysfunction. Although there are no diagnostic criteria for zinc deficiency, various methods have been proposed to diagnose it. The Japanese Society of Clinical Nutrition has published the Japanese Guidelines for the Diagnosis and Treatment of Zinc Deficiency 2018, which sets forth the diagnostic criteria for zinc deficiency [4]. That is, (a) one or more symptoms of zinc deficiency or low serum alkaline phosphatase, (b) other diseases have been ruled out, (c) serum zinc is low, and (d) symptoms are relieved by zinc administration. However, even with this criterion, as can be seen in items (b) and (d), differentiation from other diseases is difficult, and in some cases, the diagnosis can only be made after treatment. Therefore, there is a need to develop biomarkers for diagnosis. Compared to serum zinc, serum glutathione sulfotransferase omega-1 (GSTO1) responds better to zinc supplementation and has a higher correlation coefficient with zinc intake, making it promising for the diagnosis of zinc deficiency [5].

Because the zinc finger transcription factor GATA-1 is essential for both primitive and definitive erythropoiesis, zinc deficiency could cause anemia [6]. In this case, anemia that had persisted for more than a year was also observed, improved quickly with zinc supplementation, and did not recur. Zinc is crucial for the normal development and function of cells mediating non-specific immunity, such as neutrophils and natural killer cells, and zinc-deficient individuals have increased susceptibility to a variety of pathogens. In addition, zinc deficiency also affects the development of acquired immunity by preventing both the outgrowth and certain functions of T lymphocytes, and likewise, B lymphocyte development and antibody production, particularly immunoglobulin G, is compromised [7]. Her immunoglobulin G levels were slightly low, and this may have contributed to her urinary tract infection. There have been reports of cases of Clostridium difficile infection in which zinc supplementation has been found to improve symptoms and reduce recurrence rates [8]. Zinc supplementation may have contributed to the treatment of urinary tract infections and the prevention of recurrence in this case.

Zinc is essential not only for the synthesis and secretion of insulin but also for the function of insulin receptors [9]. Therefore, zinc deficiency leads to glucose intolerance [10]. In this case, even after IV treatment (Ringer's lactate), her urinary ketones were increased, and her serum glucose level was elevated. Initially, we thought that the urinary ketones reflected dehydration, but this too could have been due to zinc deficiency, and insulin resistance improved early with zinc supplementation. Zinc replacement therapy is relatively harmless, and many of its toxic effects are due to copper deficiency because zinc and copper have similar chemical properties and will compete to bind metallothionein [11]. In fact, her serum copper level declined while taking zinc acetate dihydrate but was within the normal range. Serum iron decreased with zinc supplementation, but this was a result of enhanced hematopoiesis, which improved with iron supplementation.

Zinc deficiency usually coexists with iron deficiency in women of reproductive age because they lose blood during menstruation [12]. Additionally, brown bread and milk decrease zinc absorption because phytic acid in wheat and calcium in milk form a water-insoluble complex with zinc [11]. Because the patient, who was of reproductive age, was taking iron preparations and ate pastries and milk every lunch and dinner, her serum zinc level was thought to have decreased without an iron deficiency. Since she had been on this diet for two years and her growth and bone density were normal, we determined that she began to be deficient in zinc around the same time. Adequate clinical history and dietary interview may suggest the possibility of zinc deficiency. It is also important to provide dietary guidance during treatment.

## Conclusions

Zinc deficiency is widespread but often difficult to diagnose due to the lack of specific symptoms. Since zinc deficiency is involved in various homeostases in the human body, it is very important to properly diagnose and treat it. This case is informative for the reader, as there have been few reports of patients with such a variety of symptoms and abnormal data due to zinc deficiency but who still show improvement with zinc administration.
